# Spectroscopic and Thermographic Qualities of Praseodymium-Doped Oxyfluorotellurite Glasses

**DOI:** 10.3390/molecules29133041

**Published:** 2024-06-26

**Authors:** Barbara Klimesz, Witold Ryba-Romanowski, Radosław Lisiecki

**Affiliations:** 1 Department of Physics, Opole University of Technology, Ul. Prószkowska 76, 45-758 Opole, Poland; b.klimesz@po.edu.pl; 2 Institute of Low Temperature and Structure Research, Polish Academy of Sciences, Ul. Okólna 2, 50-422 Wrocław, Poland

**Keywords:** oxyfluoride glass, Pr spectroscopy, luminescence thermometry

## Abstract

The thermal stability of oxyfluorotellurite glass systems, (65-x)TeO_2_-20ZnF_2_-12PbO-3Nb_2_O_5_-xPr_2_O_3_, doped with praseodymium was examined. The different concentrations of praseodymium oxide (x = 0.5 and 2 mol%) were applied to verify the thermal, optical and luminescence properties of the materials under study. The relatively high values of the Dietzel (ΔT) and Saad–Poulain (S or H′) thermal stability factors determined using a differential thermal analysis (DTA) indicate the good thermal stability of the glass matrix, which gradually improves with the content of the active dopant. The temperature dependence of optical spectra in the temperature range 300–675 K for the VIS–NIR region was investigated. The involved Pr^3+^ optical transition intensities and relaxation dynamic of the praseodymium luminescent level were determined. The ultrashort femtosecond pulses were utilized to examine a dynamic relaxation of the praseodymium luminescent levels. Although the measured emission of the Pr^3+^ active ions in the studied glass encompasses the quite broad spectral region, the observed luminescence may only be attributed to ^3^P_J_ excited states. As a result, the observed decrease in the experimental lifetime for the ^3^P_0_ level along with the increasing activator content was identified as an intensification of the Pr–Pr interplay and the associated self-quenching process. The maximum relative sensitivities (S_r_) estimated over a relatively wide temperature range are ~0.46% K^−1^ (at 300 K) for FIR (I_530_/I_497_) and 0.20% K^−1^ (at 600 K) for FIR (I_630_/I_497_), which seems to confirm the possibility of using investigated glasses in optical temperature sensors.

## 1. Introduction

Amorphous glass structures doped with rare earth (RE) ions attract the attention of scientists due to their wide application possibilities in optoelectronics, photonics, telecommunications, thermoelectrics, medicine and environmental protection [1,2,3,4,5,6,7,8,9]. Studies in the literature show that glasses, ceramics, fibers or crystals doped with rare earth elements can be used in various optical equipment such as solid-state lasers, broadband amplifiers, temperature sensors and fibers [10,11,12,13,14,15,16,17,18].

Moreover, in many cases, due to their unique properties as a host material, RE-doped glasses seem to be even more suitable than crystals. Glass production is easy and cheap compared to crystal. In addition, the glasses are more transparent, characterized by a high refractive index, good RE solubility, thermal stability, low phonon energy, good mechanical strength and proper non-optical linearity. They allow a relatively free selection of the chemical composition and/or its modification by various types of admixtures. This gives the possibility to influence the optical, fluorescence and melting point properties and the immediate surroundings of RE ions to a certain grade. From this point of view, it is known that the luminescence energy efficiency of rare earth ions increases if the glass matrix has low phonon energy, which is the case with tellurium glasses ≤ 800 cm^−1^ or fluoride glasses at ~500 cm^−1^ [6,19]. The main purpose is therefore to create a glass matrix whose structure will provide an appropriate environment for rare earth element admixtures to obtain the best optical output of materials depending on their applications. In this context, fluorotellurite glass combines the advantages of fluoride glass (low-energy phonon environments) with the advantages of tellurite glass (chemical durability, thermal stability and mechanical strength) [6,20,21,22].

Amongst the rare earth elements, praseodymium is a relatively alluring optical activator due to the amount of available energy levels in the UV-VIS-NIR range. Metastable states ^1^D_2_ and ^3^P_0_, when stimulated, can emit red, green and blue light simultaneously for laser operation in both crystals [10,23,24,25] and glass hosts [2,26,27,28]. And due to the ^1^D_2_ → ^1^G_4_ transition, Pr^3+^-doped glasses can also exhibit another interesting near-infrared (NIR) emission [29]. Although many spectroscopic studies for various matrices/hosts (crystals, glasses, glass ceramics) activated with Pr^3+^ ions have already been published, and optical enhancement based on ^1^G_4_ → ^3^H_5_ and ^3^F_3,4_ → ^3^H_4_ transitions in the NIR has been demonstrated, a description of the optical properties of Pr^3+^ single-doped fluorotellurite glass is still relatively rare.

Therefore, this work characterizes the variability in the spectroscopic properties of oxyfluorotellurite glass (65-x)TeO_2_-20ZnF_2_-12PbO-3Nb_2_O_5_-xPr_2_O_3_ under changes in the concentration (x = 0.5 and 2 mol%) of Pr^3+^ ions and temperature (300–675 K). The designation TZPN was adopted for the base undoped glass 65TeO_2_-20ZnF_2_-12PbO-3Nb_2_O_5_. For glasses doped with praseodymium, the designations TZPN:0.5%Pr and TZPN:2%Pr were adopted, respectively. To the best of our knowledge, the glass composition that we propose, namely (65-x)TeO_2_-20ZnF_2_-12PbO-3Nb_2_O_5_-xPr_2_O_3_ doped with praseodymium, has not yet been synthesized and/or examined. This type of amorphous material may be highly relevant owing to its still moderate red component of luminophores utilized in the light sources. The intentions of the present study are to (a) examine the impact of Pr_2_O_3_ on the oxyfluoride glass thermal properties, (b) estimate the radiative transition rates based on the modified Judd–Ofelt phenomenological theory, and (c) determine the effect of temperature on absorption and emission spectra, eventually determining the related glass thermographic qualities. Moreover, the interionic peculiarities and dynamic relaxation of the luminescent excited state were studied by employing femtosecond laser pulses.

## 2. Results and Discussion

### 2.1. Thermal Features

**Figure 1:**
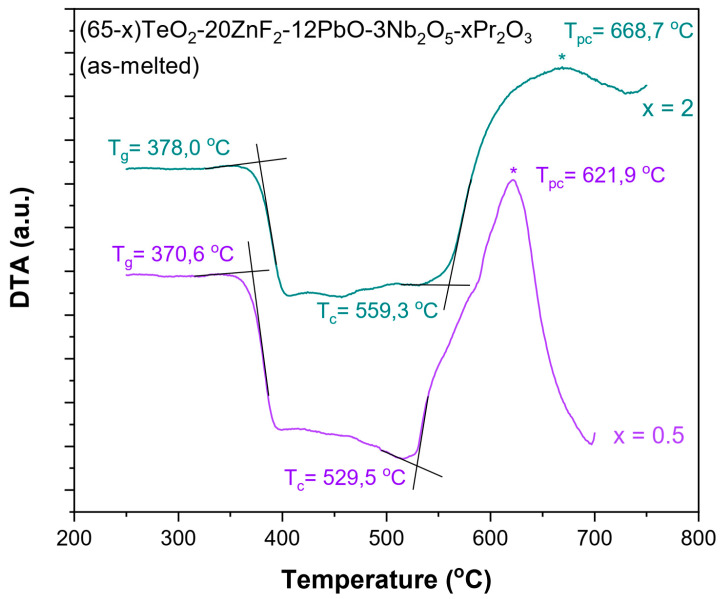
DTA curves recorded for (65-x)TeO_2_-20ZnF_2_-12PbO-3Nb_2_O_5_-xPr_2_O_3_ glasses doped with x = 0.5 and 2 mol % (asterisks indicate the maximum of peak crystallization T_pc_).

In the initial phase of the research, the thermogravimetric measurements were carried out. DTA curves were recorded for the TZPN:0.5%Pr and TZPN:2%Pr samples (Figure 1) and using the method of Keavney and Eberlin [30], characteristic temperatures were determined, such as the glass transition temperature (T_g_) and onset of crystallization temperatures (T_c_), in order to determine the thermal stability of the glasses. From the estimated data, it can be seen that the value of T_g_ indicating the initiation of the glass softening increases slightly with the concentration of Pr_2_O_3_ from 370.6 °C to 378.0 °C for samples containing 0.5 and 2 mol% Pr_2_O_3_, respectively. The T_g_ value for TZPN undoped glass is even lower and equals 365.3 °C [31]. In the case of the glass crystallization temperature, we also observe an increase in T_c_ with the Pr_2_O_3_ concentration rising. As it is seen in Figure 1, there is a shift from 529.5 °C with a 0.5 mole ratio of Pr_2_O_3_ to 559.3 °C with a 2 mole ratio of Pr_2_O_3_.

It should be noted that the exothermic crystallization peak (T_pc_) of both samples occurs in the range of 600–700 °C. Furthermore, in this case, we observe an increase in the T_pc_ with the content of the active component. A similar effect has been observed in other glass systems [9,21,32,33,34]. In the case of undoped glass 65TeO_2_-20ZnF_2_-12PbO-3Nb_2_O_5_, values of the characteristic crystallization temperatures are T_c_ = 552.3 °C and T_pc_ = 588.5 °C, respectively [31]. All this affects the ability to form the glass and its thermal stability, which can be qualitatively determined by the criteria of thermal stability:

(1) $$\Delta T=T_{c}-T_{g}\;\;\;\;\;\;\;\;\;\mathrm{Dietzel}\;\mathrm{factor},$$

(2) $$H\prime =\frac{T_{c}-T_{g}}{T_{g}}\;\;\;\;\;\;\;\;\;\mathrm{Saad}–\mathrm{Poulain}\;\mathrm{factor},$$

(3) $$S=\frac{\left(T_{c}-T_{g}\right)\left(T_{pc}-T_{c}\right)}{T_{g}}\;\;\;\;\;\;\mathrm{Saad}–\mathrm{Poulain}\;\mathrm{factor},$$

Higher values of the parameters ΔT [30], H′ [35], and S [35] mean better thermal stability of the glass and in the case of the investigated samples, their values are, respectively,

TZPN:0.5%Pr (ΔT = 158.9 °C; H′ = 0.43; S = 39.62 °C);

TZPN:2%Pr (ΔT = 181.3 °C; H′ = 0.48; S = 52.47 °C).

As it can be seen, thermal stability of the TZPN:Pr glasses improves with the content of Pr_2_O_3_. It should be noted that the estimation of the maximum T_pc_ value may be subject to a certain (difficult to eliminate) error due to the asymmetric shape of the crystallization bands (a large number of physicochemical processes take place in this temperature range), dependence location of the crystallization peak of the technical parameters of the experiment (sample mass, heating rate) and constructional features of the apparatus [36]. However, as shown in the obtained results, it does not have a major impact on the nature of changes in the thermal stability parameter S of the tested glass (it is the same trend as in the case of the parameters ΔT and H′).

### 2.2. Absorption Spectra and Modified Judd–Ofelt Analysis

**Figure 2:**
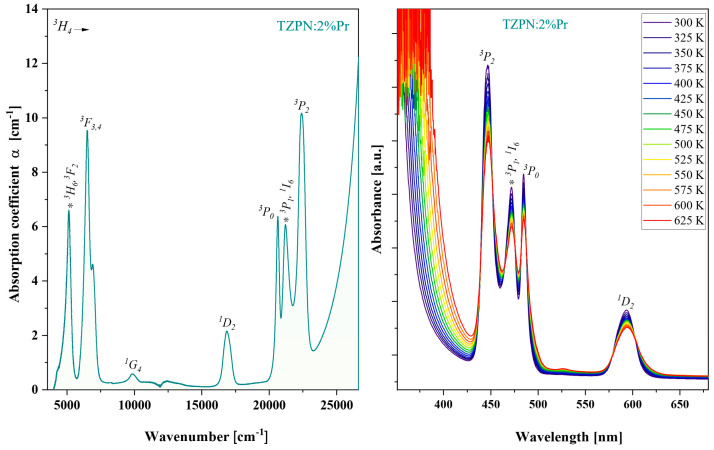
Absorption spectrum of oxyfluorotellurite glasses doped with 2%Pr (**left**). Impact of temperature, 300–625 K, on TZPN:2%Pr absorption spectra (**right**).

Absorption spectra of TZPN:Pr glass samples were investigated in the wide UV-VIS-NIR range. Figure 2 shows the absorption spectrum taken at room temperature for the 63TeO_2_-20ZnF_2_-12PbO-3Nb_2_O_5_-xPr_2_O_3_ glass sample in the wavenumber spectral range from 5000 to 25,000 cm^−1^. The visible absorption bands on the left part of Figure 2 are related to the electron transitions of praseodymium from the ground state ^3^H_4_ to respective excited states: ^3^H_6_ and ^3^F_2_ (5133 cm^−1^); ^3^F_3,4_ (6595 cm^−1^); ^1^G_4_ (9851 cm^−1^); ^1^D_2_ (16,882 cm^−1^) and groups of bands ^3^P_0,1,2_ and ^1^I_6_ (21,773 cm^−1^).

The presented absorption spectrum was used to calculate the intensity of transitions based on the Judd–Ofelt theory [37,38]. The application of the J-O theory in the case of the Pr^3+^ ion as an optical activator is not so simple and depends on the type of host matrix [10]. The absorption transitions satisfying the selection rules ΔS = 0, ΔL ≤ ±2 and ΔJ ≤ ±2 are the so-called hypersensitive transitions characterized by high values of experimental oscillator strengths [28]. The values of experimental oscillator strengths presented in Table 1 indicate that the ^3^H_4_ → ^3^P_2_ transition is hypersensitive.

Moreover, the small energy difference between the ground state configuration 4f^2^ and the first opposite parity excited configuration 4f^1^5d^1^ results in a large deviation between the measured and calculated oscillator strengths and causes some problems for fitting ^3^H_4_ → ^3^P_2_ hypersensitive transition [6]. To overcome these problems, a modified J-O theory should be used to estimate the phenomenological parameters Ω_t_.

Following the standard J-O procedure, the theoretical oscillator strengths can be calculated using the following formula:

(4) $$P_{cal}=\frac{8\pi^{2}mc}{3h\lambda \left(2J+1\right)}\frac{{\left(n^{2}+2\right)}^{2}}{9n}\times \sum_{t=2,4,6}{\Omega_{t}\left|\langle f^{N}\left[L,S\right]J\|U^{t}\|f^{N}\left[L^{\prime },S^{\prime }\right]J^{\prime }\rangle \right|}^{2}$$

where *m* is the electron mass, *c* is the speed of light, *h* is Planck’s constant, *λ* is the mean wavelength of transition, (*2J* + 1) is the degeneracy of the ground state of the lanthanide ion, $\left|\langle f^{N}\left[L,S\right]J\|U^{t}\|f^{N}\left[L^{\prime },S^{\prime }\right]J^{\prime }\rangle \right|$ are double reduced matrix elements of the unit tensor and *n* is the refractive index.

On the other hand, the experimental oscillator strengths were estimated from the absorption spectrum using the relationship

P_exp_ = 4.318 × 10^−9^∫ε(ν)dν (5)

where ε(ν) denotes the molar extinction and *ν* denotes the energy expressed in wavenumbers. Of course, the contribution of magnetic dipole transitions should also be taken into account and be subtracted from P_exp_ before the Judd–Ofelt treatment. In the case of Pr^3+^ ions, these are ^3^H_4_ → ^3^F_3,4_ and ^3^H_4_ → ^1^G_4_ transitions, respectively. Ultimately, using the least squares fitting method, this leads to the estimation of the three phenomenological parameters Ω_2,4,6_ and after obtaining them, it leads to calculating the transition rates between any given states:

(6) $$W_{r}=\frac{64\pi^{4}e^{2}}{3h\lambda^{3}\left(2J+1\right)}\frac{n{\left(n^{2}+2\right)}^{2}}{9}\sum_{t=2,4,6}{\Omega_{t}\left|\langle f^{N}\left[L\prime ,S\prime \right]J\prime \|U^{t}\|f^{N}\left[L,S\right]J\rangle \right|}^{2}$$

where *e* is the charge of the electron and all other variables have the same meaning as in the previous equations. Next, one can obtain the values of luminescence branching coefficients and radiative lifetimes using the appropriate equations:

(7) $$\beta =\frac{W_{r}\left(J^{\prime },J\right)}{\sum_{J\prime }W_{r}\left(J^{\prime },J\right)}$$

(8) $$\tau_{rad}=\frac{1}{\sum_{J\prime }W_{r}\left(J^{\prime },J\right)}$$

Unfortunately, the proposed approach in the case of praseodymium quite often, and regardless of the type of host matrix, leads to a negative value of Ω_2_ [6,10,28,39,40,41]. This requires a nonstandard approach and a certain modification of the J-O theory proposed by Kornienko [42] involving the following formula to calculate the intensity of electric dipoles:

P′_cal_ = P_cal_ × [1 + 2α (E_J_ + E_J′_ − 2E_f_^0^)] (9)

where α = ½ × [E4f5d − Ef^0^] is called the fitting parameter, which in the case of Pr^3+^ ions in any glass matrix is of the order of 1.0 × 10^−5^ cm^−1^; P′_cal_ is the oscillator force calculated from Equation (9); E_J_ is the energy of the initial state; E_J′_ is the energy of the final state; and Ef^0^ is the energy corresponding to the center of gravity of the configuration, which is ~9940 cm^−1^.

In the modified J-O model, the host-insensitive double reduced matrix elements (ǁU_t_ǁ^2^) from standard J-O theory were multiplied by the fitting parameter α. The theoretical oscillator strengths calculated in this way (P′_cal_) and experimentally determined (P_exp_) are listed in Table 1. The estimated phenomenological J-O intensity parameters (Ω_2,4,6_) are following:

Ω_2_ = 9.30 × 10^−20^ [cm^2^], Ω_4_ = 13.24 × 10^−20^ [cm^2^], Ω_6_ = 10.01 × 10^−20^ [cm^2^]

ΔΩ_2_ = 2.90 × 10^−20^ [cm^2^], ΔΩ_4_ = 0.56 × 10^−20^ [cm^2^], ΔΩ_6_ = 0.82 × 10^−20^ [cm^2^]

RMS = 1.34 × 10^−6^

The relatively small root mean square deviation (RMS = 1.34 × 10^−6^) indicates a good fit of P′_cal_ with P_exp_ and the optimal set of obtained intensity parameters: Ω_2_ = 9.30 × 10^−20^ cm^2^, Ω_4_ = 13.24 × 10^−20^ cm^2^ and Ω_6_ = 10.01 × 10^−20^ cm^2^. As it is known, there is a certain relationship between the magnitude of the phenomenological parameters Ω_2,4,6_ and the covalence of chemical bonds, structural changes in the vicinity of the incorporated rare earth ions (RE) and the bulk properties of the glass matrix [43]. There is a higher value of the Ω_2_ parameter than the greater degree of asymmetry around the Pr^3+^ ions and stronger covalence of the Pr–O bonds [6]. In turn, higher values of Ω_4_ and Ω_6_ mean lower rigidity and viscosity of the host material [10]. The trend of J-O intensity parameters observed in the TZPN:Pr glass system is Ω_2_ < Ω_6_ < Ω_4_ and is the same as in the case of other tellurite glasses doped with praseodymium [44,45,46,47]. These values are significantly higher, indicating a more asymmetric and covalent environment around Pr^3+^ ions in the tested system. In turn, the value of Ω_6_ depends more on the overlap integrals of the 4f and 5d orbits than on the environment in which the Pr^3+^ ions are located [6]. From the absorption spectra (Figure 2—right) one can notice that as the temperature increases, the absorbance of the observed absorption transitions slightly decreases without changing their position. This indicates the homogeneous distribution of Pr^3+^ ions in the glass TZPN:2%Pr and consequently, the excitation efficiency of the material under study may be insignificantly affected by the temperature elevation.

### 2.3. Emission Spectra, Radiative Properties and Color Perception

Using J-O parameters Ω_2,4,6_, the radiative properties of fluorescent transitions from ^1^G_4_, ^1^D_2_ and ^3^P_0_ levels of the studied glasses are determined. The emission performance such as radiative transition probabilities (*Wr*), luminescence branching ratios (*β*) and radiative lifetimes (*τ_rad_*) of the mentioned excited levels for the TZPN:2%Pr glass was estimated and collected in Table 2. 

The results of the J-O theory analysis fully confirm the emission spectra recorded at room temperature for TZPN:0.5%Pr and TZPN:2%Pr glass samples. Typically, with 450 nm excitation, the Pr^3+^ ions from the ^3^H_4_ ground level are excited to a higher-energy ^3^P_2_ excited state but as a result of non-radiative relaxation, a transition to the lower excited energy level ^3^P_0_ occurs. The excitation wavelength 450 nm related to a prominent ^3^H_4_ → ^3^P_2_ absorption line was selected to avoid a potential effect of the competitive processes, which might be harmful for the desired praseodymium luminescence.

**Figure 3:**
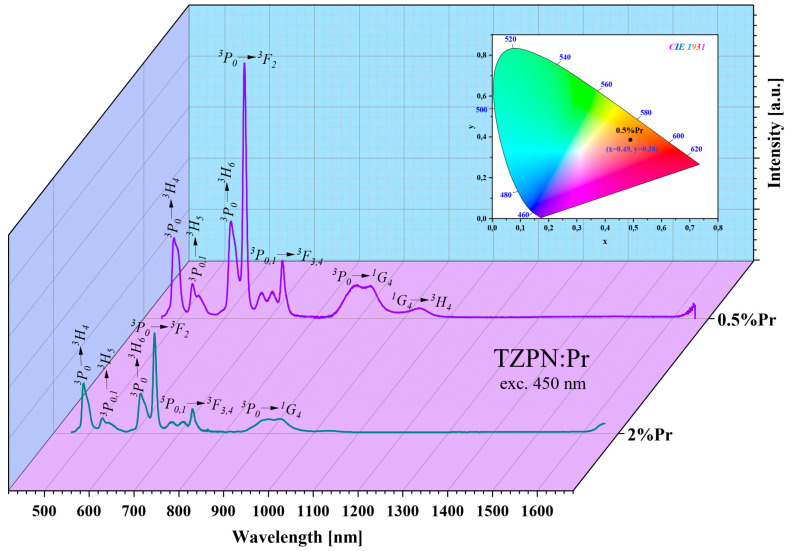
Emission spectra of TZPN:0.5%Pr and TZPN:2%Pr glasses excited at 450 nm. The inset shows a chromaticity diagram of TZPN:0.5%Pr glass luminescence.

Due to a small energy difference between the adjacent luminescent states ^3^P_0_ and ^3^P_1_, they undergo thermalization and radiative relaxation has its source in both of these excited states (^3^P_0,1_). Therefore, the emission spectra presented in Figure 3 contain bands originating mainly from the ^3^P_0_ and ^3^P_1_ excited states. In the wavelength range from 450 nm to 1100 nm, 6–7 prominent bands can be seen, where the most intense are two bands corresponding to the transitions ^3^P_0_ → ^3^H_4_ (489 nm) and ^3^P_0_ → ^3^F_2_ (645 nm).

**Figure 4:**
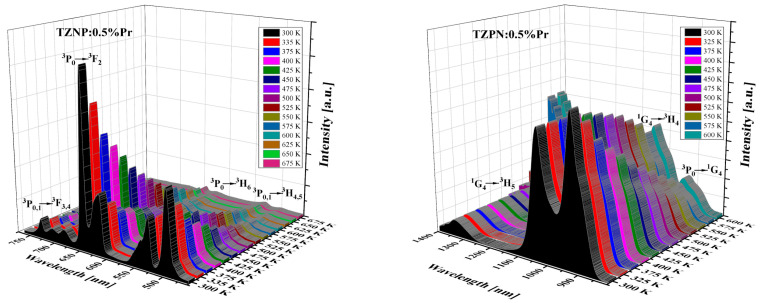
Effect of temperature on visible (**left**) and near-infrared (**right**) emission spectra of TZPN:0.5%Pr glass.

The influence of temperature on the emission spectrum of TZPN:0.5%Pr glass in the visible and near-infrared range was also examined (see Figure 4). The emission intensity of all bands decreases with increasing temperature but the spectral shape and peaks’ position are unaffected. The exception is that the band at 1327 nm corresponding to the ^1^G_4_ → ^3^H_5_ transition for that emission intensity diminishes with temperature increasing from 300 K to 600 K. The NIR luminescence at ~1.3 µm is widely used in telecommunications technology in the case of low-loss optical amplifiers operating in the O-band (1260–1360 nm) [48]. The attenuation of conventional O-band optical fiber is relatively large, for example, almost twice that of the C-band (1530–1565 nm) [49]. To compensate for this attenuation and effectively amplify light in the O-band, a praseodymium-doped fiber amplifier (PDFA) can be used based on a low-phonon-energy host glass and as one can see, the studied TZPN oxyfluorotellurite glass meets these requirements.

To determine the chromaticity of the emitted luminescence, the emission spectra profiles of TZPN glass doped with Pr^3+^ were used. The chromaticity color coordinates estimated for the TZPN:0.5%Pr glass sample excited at 450 nm are x = 0.490 and y = 0.385, respectively, and lie in the orange-red region of the chromaticity diagram of Commission Internationale de l’Eclairage (CIE) 1931 (see inset Figure 3).

The color temperature (CCT) correlated with them, determined based on the empirical McCamy formula [50]:

CCT = −449n^3^ + 3525n^2^ − 6823n + 5520.33 (10)

where n = (x − x_e_)/(y − y_e_) is the inverse slope line and x_e_ = 0.3320 and y_e_ = 0.1858 is the epicenter estimated based on the chromaticity coordinates. The CCT of TZPN:Pr oxyfluorotellurite glass at λ_exc_ = 450 nm was found to be 2128.83 K and therefore may be a promising candidate for the production of various types of photonic equipment (LEDs, solid-state lasers, displays and devices) emitting in the orange-red range of visible light [51,52].

Another important parameter due to possible luminescent applications is color purity (CP), which determines how monochromatic/pure the emitted light is and was calculated according to the relationship

(11) $$CP=\frac{\sqrt{{\left(x-x_{s}\right)}^{2}+{\left(y-y_{s}\right)}^{2}}}{\sqrt{{\left(x_{d}-x_{s}\right)}^{2}+{\left(y_{d}-y_{s}\right)}^{2}}}\times 100$$

where (x, y) are the estimated chromaticity color coordinates, (x_s_, y_s_) are the standard coordinates of white light and (x_d_, y_d_) are the coordinates of the dominant wavelength. The color purity for the glass sample TZPN:0.5%Pr reached an effective value of 45.85%. Similar CP values are reported by Poojha et al. for Pr^3+^-doped lead boro-tellurite glasses [52].

### 2.4. Photoluminescence Decay Analysis

**Figure 5:**
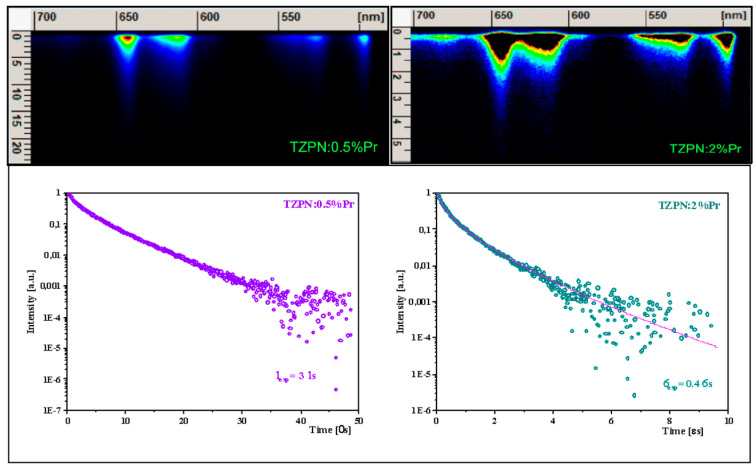
Streak camera images related to praseodymium emission in TZPN:0.5%Pr and TZPN:2%Pr glasses (**upper**) and the adequate decay curves of 3P0 luminescence (**bottom**).

The images taken from the streak camera shown in the upper part of Figure 5 consist of luminescence originating in the ^3^P_0_ excited state.

Examinations of photoluminescence (PL) decay curves for the ^3^P_0_ excited level (see lower part of Figure 5) show a partly single exponential character of the decay curve for the TZPN:0.5%Pr glass sample with a lifetime of 3 μs. When the concentration of Pr^3+^ ions increases to 2 mol%, the decay becomes faster (τ_avg_ = 0.4 μs) and the decay curve deviates from a single exponential time dependence. Consequently, we follow a commonly applied approximation and determine the so-called mean lifetime value expressed as follows [53]:

(12) $$\tau_{avg}=\frac{\int t\cdot I(t)dt}{\int I(t)dt}$$

where *t* represents time and *I* is the intensity of luminescence.

The decrease in the experimental lifetime with a higher sample concentration is related to the self-quenching effect of Pr^3+^ luminescence. This happens due to the reduced distance between Pr^3+^ ions, which in turn results in the share of non-radiative energy transfer increases through cross-relaxation and energy migration processes.

At the same time, the obtained lifetimes of the excited state ^3^P_0_ are relatively shorter than a radiative lifetime obtained by the Judd–Ofelt calculation (see Table 2). The quantum efficiency (*η*) of the tested material estimated at over 62% results from the relationship

(13) $$\eta =\frac{\tau_{exp}}{\tau_{rad}}\times 100\%$$

and in many cases is higher or comparable to the corresponding data obtained for other praseodymium-doped glasses [6,10,27,28,52,54].

The Inokuti–Hirayama model can be applied to investigate the non-radiative Pr–Pr energy transfer in material under study [55]. In the case of much slower energy migration in relation to interionic energy transfer, the time evolution of donor emission intensity may be defined as

(14) $$\Phi \left(t\right)=Aexp\left[-\left(\frac{t}{\tau_{0}}\right)-\alpha {\left(\frac{t}{\tau_{0}}\right)}^{3/S}\right]$$

where Φ(*t*) is the emission intensity after pulse excitation, *A* is constant, *S* = 6 for dipole–dipole interactions, *τ*_0_ is the intrinsic decay probability of the donor-involved excited state when the acceptor is absent and *α* is a parameter expressed as

(15) $$\alpha =\left(\frac{4}{3\pi }\right)\Gamma \left(1-\frac{3}{S}\right)N_{a}R_{0}^{3}$$

where *Na* denotes the acceptor concentration (Na = 2.36 × 10^21^), *R*_0_ is the critical Pr–Pr energy transfer distance and Γ = 1.77 (for S = 6) is Euler’s function. In fact, the recorded ^3^P_0_ decay curve for TZPN:2%Pr glass is non-exponential and as a result of the appropriate fitting, we acquired the reasonable results for S = 6 and α = 5.64. Consequently, the critical energy transfer distance was estimated to be R_0_ = 6.85 Å. An interaction parameter between praseodymium ions can be expressed as C_da_ = R_0_^6^∙τ_0_^−1^ and the related value of 2.29 × 10^−38^ cm^6^s^−1^ was estimated for our glass. Based on the formula W_da_ = Cd_a_∙R_0_^−6^, the donor–acceptor energy transfer rate was found to be 2.49 × 10^6^ s^−1^.

### 2.5. Temperature Sensor Applications

In order to assess the suitability of the studied materials for applications in optical sensing thermometry, we examined changes in the fluorescence intensity ratio (FIR) as a function of temperature (in the range of 250–700 K) and corresponding absolute (S_A_) and relative (S_R_) thermal sensitivities for TZPN:0.5%Pr glass. 

**Figure 6:**
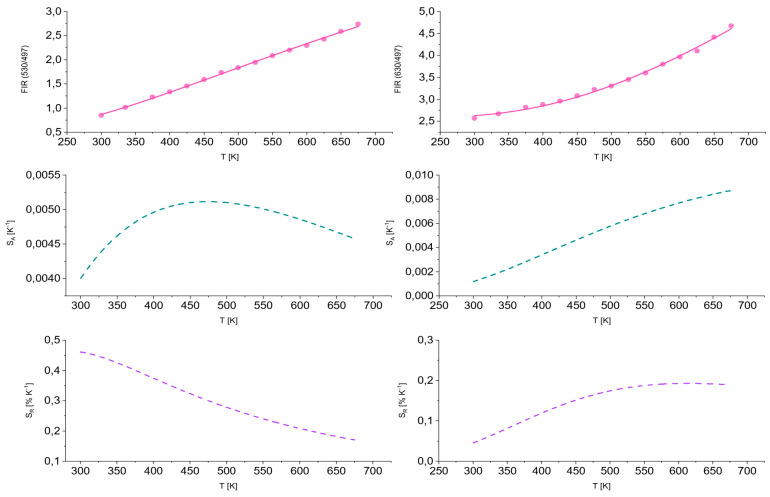
Fluorescence intensity ratios attributed to praseodymium luminescence as well as corresponding absolute and relative thermal sensitivities estimated for FIR (I_530_/I_497_) and FIR (I_630_/I_497_) for TZPN:0.5%Pr glass.

The obtained results are presented in Figure 6 in the form of temperature dependence curves of fluorescence intensity ratios FIR (I_530_/I_497_) (on the left) and FIR (I_630_/I_497_) (on the right) by fitting with the following relationship:

(16) $$FIR(T)=A+Bexp\left(\frac{\Delta E}{k_{B}T}\right)$$

where ∆*E* is the energy difference between the thermalized levels, *k_B_* is the Boltzmann constant, *T* is the temperature expressed in the absolute scale [K] and *A* and *B* are constants. In the case of FIR (I_530_/I_497_), we observe a linear increase in the fluorescence intensity ratio with a maximum absolute temperature sensitivity value of S_A_ = 5.1 × 10^−3^ K^−1^ at T = 460 K and approximately 0.46% K^−1^ relative temperature sensitivity for T = 300 K. The values of the fluorescence intensity ratios FIR (I_630_/I_497_) increase exponentially with temperature and the maximum absolute temperature sensitivity is reached for T = 675 K and amounted to 8.7 × 10^−3^ K^−1^, which translates into S_R_ = 0.20% K^−1^ of relative temperature sensitivity at about T = 460 K. All absolute and relative curves of temperature sensitivity for TZPN:0.5%Pr glass have a non-linear way with a different tendency. For a comparison, A.S. Rao examined the impact of temperature on four different emission bands of praseodymium [56]. Some fluorescence intensity ratio (FIR) models based on the relationships between different emission peaks were studied to estimate a maximum relative sensitivity, 1.03% K^−1^. Moreover, other Pr-doped inorganic phosphors were investigated by Jiawen Wang et al. [57] and a comparable evaluation approach gave rise to quite high relative sensitivity (1~3.25% K^−1^) and low temperature uncertainty (0.15–0.5 K). These reported sensitivities are higher in relation to TZPN:Pr estimations but regardless, our results are attributed to a wide temperature range up to 675 K.

## 3. Materials and Methods

A traditional melt quenching method was employed to manufacture TeO_2_-ZnF_2_-PbO-Nb_2_O_5_ oxyfluoride glasses activated with 0.5 and 2 mol% of Pr_2_O_3_. Tellurium oxide (5N), zinc fluoride (5N), lead oxide (4N), niobium oxide (Nb_2_O_3_) and praseodymium oxide (5N) were thoroughly mixed, ground and incorporated in a corundum crucible. The melting process in the ambient atmosphere took 30 min at 830 °C. The glasses were poured into preheated brass molds and then annealed for a few hours at 350 °C in order to reduce the internal stresses. The resulting glass samples were transparent and homogeneous.

The specimens were characterized by the DTA (differential thermal analysis) technique applying the normal pressure and atmosphere. The DSC 404/3/F calorimeter (Erich NETZSCH B.V. & Co. Holding KG Gebrüder-Netzsch, Selb, Germany), platinum crucibles and reference holders were adequately employed. The same heating rates (10 K/min) were realized for all studied glasses.

The spectrophotometer Agilent Cary 5000 (Agilent, Santa Clara, CA, USA) was used to measure the survey absorption spectra within UV-VIS-NIR spectral ranges. Luminescence experiments were carried out in the visible and near-infrared spectral regions as well. An FLS1000 Spectrofluorimeter (Edinburgh Instruments Ltd., Livingston, UK) was utilized to record the emission spectra. The Linkam THMS 600 Heating/Freezing Stage (Linkam Scientific Instruments Ltd., Redhill, UK) was used to perform temperature-dependent measurements. To record luminescence decay curves, the glass samples were excited by a femtosecond LIBRA Ti:sapphire laser (Coherent Inc., Santa Clara, CA, USA) coupled with optical parametric amplifier “Opera” (OPO).

## 4. Conclusions

It was found that TZPN glass thermal stability increases for a higher concentration of optically active ions. The impact of temperature on the absorption and variation of fluorescence intensity in the VIS-NIR region was monitored in the temperature range of 300–675 K. The observed luminescence can be mainly attributed to transitions originating from two closely located, thermally coupled levels (^3^P_0_ and ^3^P_1_). The values of the radiative transition probabilities Wr, branching ratios β and the fluorescence intensity ratio at different temperatures for the selected praseodymium transitions were adequately investigated. The branching ratio of praseodymium luminescence in material under study is related to a wide spectral region; hence, its various useful potential applications can be considered. The CIE coordinates for the TZPN:Pr glasses are in the orange-red region. The maximum lifetime of the ^3^P_0_ level was observed for a sample with a lower concentration of active ions. This is explained by the self-quenching effect—the experimental lifetime decreases with increasing Pr^3+^ concentration. Application potential of the investigated material in optical sensor thermometry was evaluated as well.

## Figures and Tables

**Table 1:** Oscillator strengths of Pr^3+^ f-f transitions in 63TeO_2_-20ZnF_2_-12Pb_2_O_5_-3Nb_2_O_5_-2Pr_2_O_3_ at 300 K.

Transition ^3^H_4_→	Energy ν [cm^−1^]	Oscillator Strengths P × 10^−6^	
		P_exp_	P′_cal_
^3^H_6_, ^3^F_2_	5133	9.74	9.72
^3^F_3_, ^3^F_4_	6595	20.44	20.70
^1^G_4_	9851	0.57	0.70
^1^D_2_	16,882	4.78	2.91
^3^P_0_, ^3^P_1_, ^1^I_6_, ^3^P_2_	21,773	39.55	39.55

**Table 2:** Calculated values of the radiative transition rates *W_r_*, luminescence branching ratios *β* and total radiative lifetimes *τ_rad_* for excited states of Pr^3+^ in 63TeO_2_-20ZnF_2_-12PbO-3Nb_2_O_5_-2Pr_2_O_3_.

*SLJ*	*S*′*L*′*J*′	Average Wavelength [μm]	*W_r_* [s^−1^]	*β*	*τ_rad_* [μs]
^1^G_4_	^3^H_4_	1.037	209.43	1.0000	4774.78
	^3^H_5_	1.327	0.00	0.0000	
	^3^H_6_	1.868	0.00	0.0000	
	^3^F_2_	2.124	0.00	0.0000	
	^3^F_3,4_	3.082	0.00	0.0000	
^1^D_2_	^3^H_4_	0.600	4124.10	0.2521	61.13
	^3^H_5_	0.687	101.56	0.0062	
	^3^H_6_	0.808	2083.52	0.1274	
	^3^F_2_	0.853	2324.58	0.1421	
	^3^F_3,4_	0.974	7724.73	0.4722	
	^1^G_4_	1.427	0.00	0.0000	
^3^P_0_	^3^H_4_	0.489	110,347.25	0.5309	4.81
	^3^H_5_	0.545	0.00	0.0000	
	^3^H_6_	0.619	17,288.85	0.0832	
	^3^F_2_	0.645	57,824.60	0.2782	
	^3^F_3,4_	0.711	22,341.41	0.1075	
	^1^G_4_	0.927	0.00	0.0000	
	^1^D_2_	2.661	42.36	0.0002	

## Data Availability

Data are contained within the article.
